# *In Vitro* Synthesized RNA Generated from cDNA Clones of Both Genomic Components of *Cucurbit yellow stunting disorder virus* Replicates in Cucumber Protoplasts

**DOI:** 10.3390/v8060170

**Published:** 2016-06-14

**Authors:** Carolyn A. Owen, Romy Moukarzel, Xiao Huang, Mona A. Kassem, Eleonora Eliasco, Miguel A. Aranda, Robert H. A. Coutts, Ioannis C. Livieratos

**Affiliations:** 1 Department of Sustainable Agriculture, Mediterranean Agronomic Institute of Chania, Alsylio Agrokepio, Chania GR-73100, Greece; owen@maich.gr (C.A.O.); moukarzel.romy@gmail.com (R.M.); 2 Sir Alexander Fleming Building, Department of Biological Sciences, Imperial College, London SW7 2AZ, UK; huang.xiao@gmail.com (X.H.); eeliasco@hotmail.com (E.E.); 3 Departamento de Biología del Estrés y Patología Vegetal, Centro de Edafología y Biología Aplicada del Segura (CEBAS)-CSIC, P.O. Box 164, 30100 Espinardo, Murcia, Spain; mona_kassem@yahoo.com (M.A.K.); m.aranda@cebas.csic.es (M.A.A.); 4 Centro de Investigacion en Quimica Aplicada, CIQA-CONACYT, Saltillo 25294, Mexico; 5 Department of Biological and Environmental Sciences, School of Life and Medical Sciences, University of Hertfordshire, College Lane, Hatfield, Hertfordshire AL10 9AB, UK; r.coutts@herts.ac.uk

**Keywords:** criniviruses, whitefly-transmitted viruses, *Cucurbit yellow stunting disorder virus*, infectious clones, protoplasts

## Abstract

*Cucurbit yellow stunting disorder virus* (CYSDV), a bipartite whitefly-transmitted virus, constitutes a major threat to commercial cucurbit production worldwide. Here, construction of full-length CYSDV RNA1 and RNA2 cDNA clones allowed the *in vitro* synthesis of RNA transcripts able to replicate in cucumber protoplasts. CYSDV RNA1 proved competent for replication; transcription of both polarities of the genomic RNA was detectable 24 h post inoculation. Hybridization of total RNA extracted from transfected protoplasts or from naturally CYSDV-infected cucurbits revealed high-level transcription of the p22 subgenomic RNA species. Replication of CYSDV RNA2 following co-transfection with RNA1 was also observed, with similar transcription kinetics. A CYSDV RNA2 cDNA clone (T3CM8Δ) comprising the 5′- and 3′-UTRs plus the 3′-terminal gene, generated a 2.8 kb RNA able to replicate to high levels in protoplasts in the presence of CYSDV RNA1. The clone T3CM8Δ will facilitate reverse genetics studies of CYSDV gene function and RNA replication determinants.

*Cucurbit yellow stunting disorder virus* (CYSDV; genus *Crinivirus*) is among the most important cucurbit pathogens worldwide, causing diseases with serious economic impact [1,2]. CYSDV is efficiently transmitted by two of the most invasive species of the *Bemisia tabaci* complex, the Mediterranean (MED, previously referred to as biotype Q) and the Middle East-Asia Minor 1 (MEAM1, biotype B). As both MED and MEAM1 are now distributed throughout the sub-tropical and temperate regions where cucurbit cultivation chiefly occurs, their ubiquity has been a major factor in the emergence and spread of a number of important viral pathogens including CYSDV [3]. Because criniviruses cannot be mechanically transmitted, full-length infectious cDNA clones are essential for the experimental infection of model plants in the absence of whiteflies and for the creation and propagation of recombinant viruses for reverse genetics studies to examine the function(s) of viral sequences and RNA structures.

The large size and complexity of crinivirus genomes, and the propensity of their cDNA clones to rearrange or to cause toxicity in bacteria has limited the production of infectious clones to only three criniviruses. In the first instance infectious transcripts of the genus type species *Lettuce infectious yellows virus* (LIYV), with expression of the viral sequences driven by bacteriophage RNA polymerase promoters, were used to transfect tobacco protoplasts [4]. These clones, in conjunction with later versions permitting *Agrobacterium*-mediated transformation of plants with LIYV cDNAs under the control of the CaMV 35S promoter [5], generated important insights into LIYV biology [6]. It was shown that RNA1 is competent to replicate alone [4], and to drive the *trans*-replication of LIYV RNA2 in an asynchronous manner [7]. The RNA1-encoded p34 was shown to be essential for RNA2 replication [7], and to bind single stranded RNA [8]. The RNA2-encoded heat shock protein 70 homologue (Hsp70h), p59, CP, and CPm were shown to be virion structural proteins [9], with CPm being the sole determinant of whitefly transmission [9,10,11]. *Lettuce chlorosis virus* (LCV) infectious clones [12,13] have also revealed further aspects of crinivirus biology, including the synchronous replication of LCV RNA1 and RNA2 molecules, and the existence of defective RNAs arising from the former. Shifts in vector population dynamics have greatly reduced the agricultural threat from LIYV, and LCV has little economic impact on agricultural production. Therefore research is now focused on the more economically important criniviruses [2]. Recently, infectious clones were reported for *Tomato chlorosis virus* (ToCV), a crinivirus responsible for severe damage to tomato production that also infects other important crop species including sweet pepper and potato [14]. Here, the first CYSDV infectious clones able to replicate in cucumber protoplasts are described.

Throughout, detection of the genomic (g) RNA species and co-terminal subgenomic (sg) RNAs derived from RNA1 and RNA2 was conducted by hybridisation of northern blots. The dig-labelled riboprobes used were transcribed from cloned cDNAs encoding p22 and p26, the most 3′-proximal genes of CYSDV RNA1 and RNA2, respectively. Figure 1A shows the hybridisation patterns obtained from RNA extracted from CYSDV-infected cucumber leaves from the field, using p22 (left panel), and p26 (right panel), negative (−) sense riboprobes. Each probe hybridised to the corresponding gRNA, plus a number of smaller species. The smallest and most prominent RNA species detected in the RNA1 hybridisation was determined to be the p22 sgRNA on the basis of its size (*ca.* 900 bp).

A full length cDNA clone of CYSDV gRNA1 (9127 bp) was constructed by amplifying a series of seven overlapping cDNA fragments from CYSDV dsRNA using *Pfu* polymerase (Promega, Madison, WI, USA), with each pair of adjacent fragments sharing a unique restriction site occurring in the sequence. The first PCR primer introduced a *Not*I site to facilitate cloning and a T7 polymerase binding site immediately upstream of the viral 5′-UTR. A G/C substitution was made at the final nucleotide of the 3′-UTR to introduce a terminal *Xho*I site to allow generation of a linear transcription template. The restricted PCR fragments were serially cloned as double ligation pairs into the plasmid vector pBluescript, to produce pBS_T7CYSDVRNA1. The insert was sequenced, and the purified linearised construct was used as a template to produce capped mRNA transcripts using the mMessage mMachine system (Thermo Fisher Scientific, Waltham, MA, USA).

CYSDV RNA1 transcripts were tested for replication competence in *Nicotiana benthamiana* mesophyll protoplasts, using a polyethyleneglycol (PEG)-mediated transfection protocol, as described by Mathioudakis *et al.* [15]. However, in repeated experiments, in which 10 μg CYSDV RNA1 capped transcripts were introduced per million protoplasts, no replication was detected. While *N. benthamiana* is not a natural host for CYSDV, the RNA1 of the criniviruses LIYV, ToCV, and the phylogenetically close relative of CYSDV, LCV, all have been demonstrated to replicate in protoplasts of this species [4,13,14]. Similarly, for the closterovirus *Citrus tristeza virus* (CTV), replication of virion RNA in *N. benthamiana* protoplasts was more pronounced than in those isolated from its natural woody host *Citrus sinensis* [16].

The CYSDV RNA1 construct was then tested in cucumber (*Cucumis sativus* c.v. Ravenna) first-leaf mesophyll protoplasts, isolated by the same procedure as for the *N. benthamiana* protoplasts but with the centrifugation force increased to 190× *g* to compensate for the smaller cell size. The cucumber protoplasts were less robust than those of *N. benthamiana* and were difficult to maintain in culture beyond 72 h, but following transfection RNA1 replication was detected at 36 h post inoculation (p.i.), and was further increased at 72 h (Figure 1B).

In time-course experiments, the input mRNA was detectable on inoculation but had reduced to undetectable levels after 12 h (Supplementary Figure S1), while bands corresponding to *de novo* synthesized positive strand gRNA and the p22 sgRNA were detected 24 h p.i. The amounts of both species increased further at 48 h and remained high at 72 h p.i. (Figure 1C upper panel). No alteration in the relative intensity of the two species was observed throughout the time course, and the hybridisation pattern was the same as that seen from infected plant tissue. The intensity of the two bands was approximately equal; as their respective sizes are 9.1 kb and 0.9 kb, transcription of the p22 sgRNA is proportionally greater. On longer exposures, faint bands corresponding to RNA species of *ca.* 1600 bp, and 1400 bp were visible; these, on the basis of their sizes were determined to be the 3′-co-terminal sgRNAs for CYSDV p5 and p25, respectively. The prominent p22 sgRNA band was also detected consistently in RNA isolated from different CYSDV-infected melon and cucumber samples, indicating the absence of temporal regulation of p22 sgRNA transcription in natural infections, as well as in transfected protoplasts. Analogous results have also been reported for the p34 sgRNA of LIYV [4,7] and the p23 sgRNA of LCV [13], which are also transcribed from the most 3′-proximal coding sequence of each respective crinivirus RNA1.

On a duplicate blot hybridised with a p22 positive sense (+) probe, a single band corresponding to the negative sense RNA1 was detected that first appeared at 24 h, increased in intensity at 48 h, and remained high at 72 h p.i. (Figure 1C middle panel). The CYSDV gRNA1 (−) strand hybridisation signal was weaker than that seen with the equivalently-labelled p22 (−) probe, requiring longer exposures to reveal the bands, indicating that the replication of CYSDV RNA1 is asymmetric, with accumulation of CYSDV gRNA1 (+) strand being greater than that of the (−) strand (Figure 1C).

Crinivirus genomes exhibit a generally conserved architecture, while each has unique features [1,6]. Interspecies variation is seen in RNA 1 downstream of RdRp, where 1–3 ORFs occur, for which the predicted amino acid homology among the proteins encoded by similarly-sized and equivalently-positioned genes is low. For three criniviruses, proteins encoded by genes in this region of RNA1 have been reported to be suppressors of RNA silencing: *Sweet potato chlorotic stunt virus* (SPCSV) p22 [17], ToCV p22 [18] and CYSDV p25 [19]. In SPCSV the p34 gene encodes an RNase type III protein bearing no resemblance to any other viral protein, that has been shown to enhance the RNA silencing suppression activity of p22 [17], and to mediate synergism between SPCSV and *Sweet potato feathery mottle virus* (SPFMV) [20]. ToCV p22 has been recently shown to preferentially bind dsRNA species and to protect them from degradation [21]. In CYSDV the p22 gene exhibits no homology with any characterised protein and has yet to have any function ascribed to it. The early and sustained transcription of the p22 sgRNA in transfected protoplasts, and its maintained presence in natural CYSDV infections, suggests its importance for the initiation and promotion of infection and underlines the necessity of further investigations to characterise its function(s) and intracellular localisation.

To construct a full-length cDNA clone for CYSDV gRNA2 (7976 bp), two overlapping cDNA fragments were amplified from total RNA extracted from CYSDV-infected plants using *Pfu* polymerase (Promega). Overlapping PCR was used to produce full-length cDNAs, which were cloned into pGEM-T-Easy vector (Promega). A blunt-ended (Q5; NEB, Ipswitch, MA, USA) PCR product amplified from one RNA2 clone (pGEMT_CM8), using primers corresponding to the natural ends of the CYSDV RNA2 sequence that introduced a T3 RNA polymerase site 5′ of the viral sequence, was used as the template to produce capped mRNA transcripts. On transfection of 10 μg CYSDV RNA2 mRNA alone into protoplasts no replication was detected. However, upon co-transfection with equimolar RNA1 mRNA (10 μg RNA1, 8.8 μg RNA2/10^6^ protoplasts), replication of RNA2 was observed. The full-length positive-strand CYSDV RNA2 was detectable in total protoplast RNA extracts hybridised with the CYSDV p26 (−) riboprobe at 24 h, significantly increased in intensity at 48 h and remained elevated at 72 h p.i. (Figure 2). A number of less intense bands corresponding to smaller RNA species could be observed at the limit of detection. These presumably correspond to 3′ co-terminal sgRNAs, as at least six such species have been predicted to arise from CYSDV RNA2 [22]. The ability of CYSDV RNA1, and not RNA2, to replicate alone in protoplasts indicates that as for LIYV, LCV and ToCV [7,13,14] the latter RNA does not encode any proteins required for virus replication. Attempts to RT-PCR amplify *de novo* defective 5′- and 3′-co-terminal CYSDV RNA2 species analogous with those described by Rubio and co-workers [23,24] from transfected protoplasts were unsuccessful.

The relative expression patterns of CYSDV RNA1 and RNA2, recall the virtually synchronous replication observed for LCV RNA1 and RNA2 in tobacco protoplasts transfected with either virions [25], or infectious RNA transcripts [13]. These results contrast with those seen for LIYV, where transfection of virion RNA into tobacco protoplasts resulted in 24 h delays in the initiation and attainment of maximal replication of RNA2, relative to RNA1 [7]. This indicates that for CYSDV, as for LCV, RNA2 transcription is effected by RNA1, with a minimal lag period in its appearance. Folding-analysis of the 3′-UTR regions of criniviruses predicts the presence of characteristic stem loop structures and terminal pseudoknots that have been shown in other viral sequences to be important for the initiation of (−) strand RNA synthesis [26]. Comparison of the 3′-UTR regions of RNA1 and RNA2 from CYSDV, LIYV, and SPCSV revealed conserved predicted secondary structure in all cases, except for LIYV RNA2 which lacked one of the four hairpin loops and the pseudoknot structure [27]. This variation might account for the similar replication kinetics of LCV and CYSDV RNA1 and RNA2, and the delay in RNA2 synthesis for LIYV.

An artificial defective CYSDV RNA2 cDNA clone was created by *Bgl*II restriction and re-ligation of pGEMT_CM8 to excise nucleotides 1116-6325 of RNA2. Following PCR amplification (LA Taq; Clontech, Pala Alto, CA, USA) of the 2.8 kbp modified sequence using primers that introduced a T3 polymerase binding site 5′ of the viral sequence and an *Nru*I site to allow restriction at its natural end, the product was cloned as pGEMT_T3CM8Δ. This construct served as a template for the production of a capped mRNA transcript, RNA2Δ, shown in schematic representation in Figure 3A, which comprises 95% of the 5′-UTR, the distal half of the CPm gene, the p26 gene and the 3′-UTR of RNA2. When transfected into cucumber protoplasts with equimolar RNA1 (10 μg RNA1, 3.1 μg RNA2Δ/10^6^ protoplasts), RNA2Δ accumulated to high levels with similar kinetics to CYSDV RNA2 (Figure 3B). No replication was seen in the absence of RNA1. Thus, the last 62 nucleotides (1116–1177) of the CYSDV RNA2 5′-UTR, the six RNA2-encoded proteins that were deleted (ORFs5-10: p5, Hsp70h, p6, p59, p9, CP), and the partially deleted CPm, are all dispensable for CYSDV RNA2 replication. Previously, mutation studies conducted on LIYV had demonstrated that the individual elimination of the five largest RNA2-encoded proteins (Hsp70h, p59, CP, CPm, and p26) via the introduction of stop codons to abort translation did not affect the accumulation of either RNA1 or RNA2, indicating that none is required for LIYV RNA2 replication [7]. Much shorter exposure times were required to reveal the RNA2Δ band in comparison with that from RNA2, indicating higher levels of replication of the former. Similarly, it has previously been shown for the (unipartite genome) closteroviruses *Beet yellows virus* (BYV) and CTV, that cDNA clones in which all non-replicase genes were deleted generated RNA that replicated to higher levels than full-length constructs in *N. benthamiana* protoplasts [28,29].

A number of features of the T3CM8Δ clone make it a useful tool to define the 3′- and 5′-UTR sequences required for the replication of CYSDV RNA2: its small size allows ready amplification and easy purification, and makes it an ideal template for the rapid generation of mutants using high-fidelity PCR-driven mutation strategies. The replication of its RNA transcript to high levels within 36 h in protoplasts will facilitate rapid simultaneous evaluation of panels of mutant defective RNAs without recourse to whole plant studies. Introduction of a reporter gene such as that encoding green fluorescent protein immediately downstream of the 5′-UTR could obviate the need for northern hybridization by allowing the photometric quantitation of the replication of RNA2 mutants and dissection of translation-essential domains within the exceptionally long 5′-UTR which, at 1177 bp, is considerably longer than that of any other known plant virus [6,22].

CYSDV is a prominent crinivirus exerting a major negative impact on cucurbit production in several areas of the world, a phenomenon exacerbated by the lack of effective whitefly control measures. It is anticipated that containment of CYSDV and other whitefly-associated plant viruses will be most easily achieved via the development of resistant plant varieties or mechanisms to block virus transmission [1]. Central to these aims are the creation of genetic tools to allow dissection of plant-virus [14], virus-vector [10,30] and vector-plant [31] interactions at a molecular level. The construction of CYSDV cDNA infectious clones represents a first step towards this goal.

## Figures and Tables

**Figure 1:**
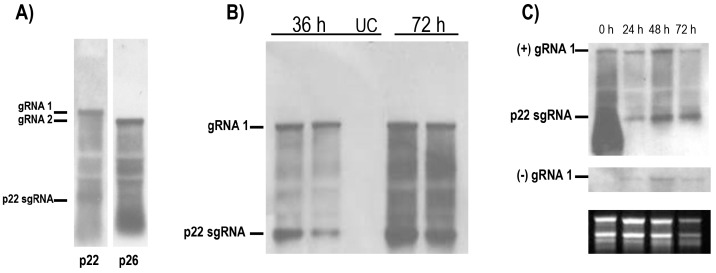
CYSDV RNA1 replicates independently of RNA2 in cucumber protoplasts. Positive and negative strand accumulation occurs with similar kinetics, but with quantitative asymmetry. (**A**) Northern blots of total RNA extracted from field samples of naturally CYSDV infected cucumber plants showing gRNA and positive co-terminal RNA species hybridising with a (−) sense p22 probe (left panel, RNA1), and a (−) sense p26 probe (right panel, RNA2). The position of the sgRNA for CYSDV p22 is indicated; (**B**) Northern blot of total RNA extracted 36 h and 72 h p.i. from protoplasts transfected in conjunction with CYSDV RNA1, hybridised with a (−) sense p22 probe. UC = uninfected control; (**C**) Time course of RNA1 transfected protoplasts hybridised with (−) and (+) sense probes. Duplicate northern blots were hybridised with dig-labelled riboprobes corresponding to the (−) sense (top panel) and (+) sense (middle panel) of the CYSDV p22 gene. The lower panel shows the EtBr stained loading controls. The positions of the gRNA bands and the p22 sgRNA are indicated.

**Figure 2:**
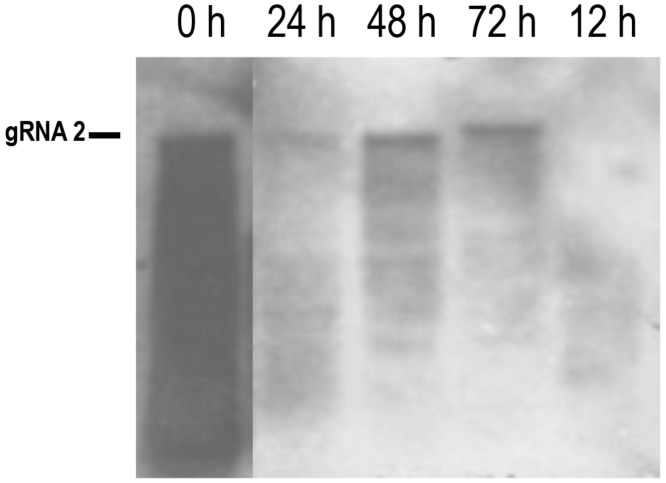
Replication of CYSDV RNA2 occurs in the presence of RNA1, with similar kinetics and a small temporal delay. A time-course northern blot of total RNA extracted from protoplasts co-transfected with equimolar amounts of CYSDV RNA1 and RNA2, hybridised with a dig-labelled riboprobe corresponding to the (−) sense CYSDV p26 gene. The position of the RNA2 gRNA is indicated. The apparent shift in the gRNA band at 72 h p.i. is an electrophoresis artefact.

**Figure 3:**
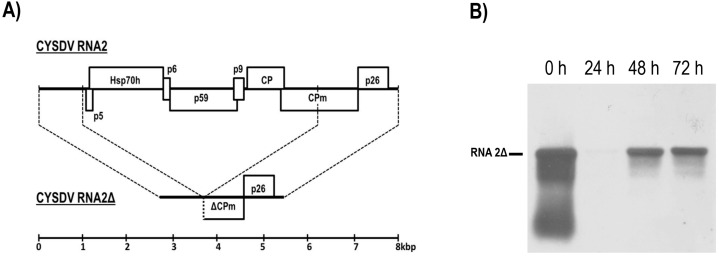
An artificial defective RNA2 transcript replicates in cucumber protoplasts in the presence of RNA1. (**A**) Schematic showing the relationship between CYSDV RNA2 and RNA2Δ, a deletion mutant created by removing nucleotides 1116–6325 of CYSDV RNA2; (**B**) a time-course northern blot of total RNA extracted from the protoplasts co-transfected with equimolar amounts of CYSDV RNA1 and RNA2Δ, hybridised with a dig-labelled riboprobe of the (−) sense CYSDV p26 gene. The position of RNA2Δ is indicated.

## Supplementary Materials

Supplementary File 1
