# Supplementary material for: In Vitro Synthesized RNA Generated from cDNA Clones of Both Genomic Components of Cucurbit yellow stunting disorder virus Replicates in Cucumber Protoplasts
Source: Viruses. 2016 Jun 14;8(6):170. doi: 10.3390/v8060170 (PMC4926190; doi:10.3390/v8060170)
Supplement: Supplementary File 1 [file viruses-08-00170-s001.pdf]

Supplementary Materials: *In Vitro* Synthesized RNA Generated from cDNA Clones of Both Genomic Components of *Cucurbit yellow stunting disorder virus* Replicates in Cucumber Protoplasts

Carolyn A. Owen, Romy Moukarzel, Xiao Huang, Mona A. Kassem, Eleonora Eliasco, Miguel A. Aranda, Robert H.A. Coutts and Ioannis C. Livieratos

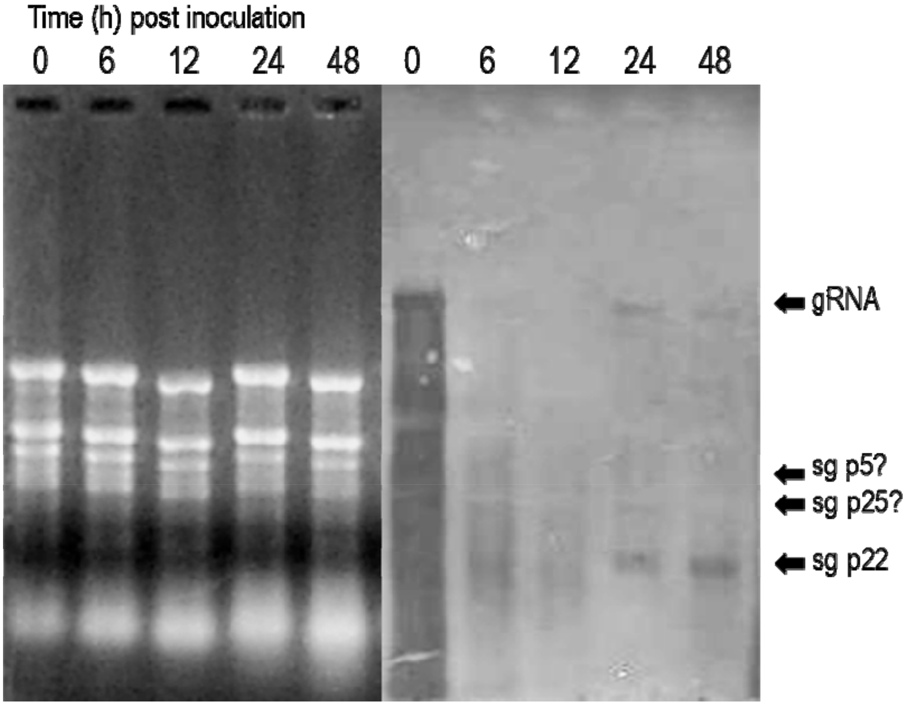

Figure S1. Input mRNA in *C. sativus* protoplasts transfected with CYSDV RNA1 mRNA is reduced to undetectable levels at 12 h post-transfection, while *de novo*-synthesised CYSDV RNA1 is first detectable at 24 h p.i.. Northern blot of total protoplast RNA isolated at the indicated time points hybridised with the p22 negative (−) sense riboprobe. Extended exposures did not reveal the presence of full-length inoculate at 12 h p.i..

© 2016 by the authors; licensee MDPI, Basel, Switzerland. This article is an open access article distributed under the terms and conditions of the Creative Commons by Attribution (CC-BY) license (http://creativecommons.org/licenses/by/4.0/).
